# Clinical implication of ANTWERP score in patients receiving catheter ablation for atrial fibrillation

**DOI:** 10.1002/joa3.13193

**Published:** 2024-11-25

**Authors:** Naoya Kataoka, Teruhiko Imamura

**Affiliations:** ^1^ Second Department of Internal Medicine University of Toyama Toyama Japan

To editor:

Catheter ablation for atrial fibrillation (AF) in patients with systolic heart failure is a well‐established therapeutic procedure. However, predicting the degree of improvement in left ventricular ejection fraction (LVEF) following catheter ablation remains challenging. Ling and colleagues recently applied the ANTWERP score, a novel predictive tool, to estimate postablation LVEF improvement in an Asian cohort.^1^ They determined a specific ANTWERP score cutoff to identify “responders” — patients who are likely to experience significant LVEF enhancement after ablation. Nonetheless, several concerns warrant attention.

The clinical utility of the ANTWERP score for assessing catheter ablation candidacy in AF patients remains ambiguous. For instance, recent studies have introduced a novel classification for response to cardiac resynchronization therapy (CRT). Following CRT initiation, patients exhibiting early LVEF stabilization demonstrate superior clinical outcomes compared to those with subsequent LVEF decline.^2^ Notably, an LVEF increase is not a requisite for classifying responders to CRT. Similarly, in the context of AF catheter ablation, LVEF improvement may be limited in patients with a history of myocardial infarction. Nevertheless, ablation remains indicated in such cases to prevent heart failure exacerbation. Furthermore, catheter ablation may also be indicated to reduce AF burden, potentially leading to LVEF improvement, regardless of the ANTWERP score. Thus, ablation could be justified in patients with reduced LVEF irrespective of their ANTWERP score.

The ANTWERP score does not appear to predict AF recurrence postablation effectively.^1^ In their study, nonresponders experience early AF recurrence, whereas responders are more likely to encounter recurrence beyond 1 year postprocedure. Nonresponders with elevated ANTWERP scores frequently exhibited larger left atria and pulmonary veins, which may facilitate reconnection gaps. In contrast, late‐phase AF recurrence in responders may stem from nonpulmonary vein foci.^3^ Did the authors collect data regarding the specific origins of AF recurrence? Tailoring the therapeutic approach for AF catheter ablation may benefit from stratification by ANTWERP score.

## CONFLICT OF INTEREST STATEMENT

Authors declare no conflict of interests for this article.
